# 1,5-Disubstituted Tetrazoles as Promising Anticancer Resistance Agents: A Chemoinformatic Characterization

**DOI:** 10.1021/acsomega.6c00605

**Published:** 2026-05-20

**Authors:** Camila Garibay-Manríquez, Erik Díaz-Cervantes, Luis Chacón-García, Karina Martinez-Mayorga, Carlos J. Cortés-García

**Affiliations:** † Universidad Michoacana de San Nicolas de Hidalgo Instituto de Investigaciones Quimico Biologicas, Laboratorio de Diseño de Molecular Ciudad Universitaria Morelia, Michoacán 58030, Mexico; ‡ Instituto de Química, Unidad Mérida, Universidad Nacional Autónoma de México, Carretera Mérida-Tetiz, Km. 4.5, Ucú, Yucatán 97357, Mexico; § Departamento de Alimentos, Centro Interdisciplinario del Noreste, Universidad de Guanajuato, Tierra Blanca, Guanajuato 37975, Mexico

## Abstract

The 1,5-disubstituted tetrazole (1,5-DS-T) scaffold is recognized as a privileged structure in medicinal chemistry due to its broad interaction potential with biologically relevant targets. In this study, we conducted an integrated chemoinformatic and molecular modeling analysis of 1,5-DS-T compounds from PubChem, ChEMBL, DrugBank, and an in-house library. Principal component analysis and scaffold diversity analyses showed that the in-house compounds occupy a region of chemical space largely overlapping with bioactive ChEMBL tetrazoles, while also revealing opportunities for further scaffold diversification. Analysis of pIC_50_ values and annotated therapeutic targets highlighted the broad bioactivity profile of 1,5-DS-T across pharmacologically relevant protein classes. Ligand-based target prediction combined with *k*-means clustering suggested CXCR3, a chemokine receptor implicated in cancer drug resistance, as a plausible target for the in-house collection. Subsequent molecular docking studies prioritized **IH-114** as the most promising computational hit, and Boltz-2 analysis supports the proposed CXCR3 binding. Finally, we found that only a small fraction of bioactive 1,5-DS-T compounds were synthesized via the Ugi-azide reaction, a modular chemical strategy, central to our research work. This highlights the need for further exploration and synthesis of new 1,5-disubstituted tetrazole-based scaffolds via isocyanide-based multicomponent reactions to explore new regions of the chemical space and discover novel drug candidates targeting chemoresistant diseases.

## Graphical abstract

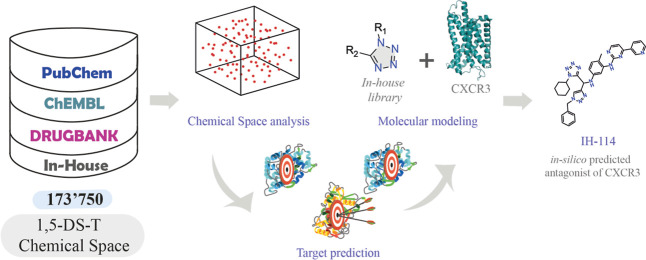

## Introduction

Drug resistance represents a significant health burden due to its occurrence across multiple diseases, most notably in cancer and microbial infections.^1,2^ The failure to effectively address drug resistance in these conditions has serious consequences, including a growing number of annual deaths. Antimicrobial bacterial resistance (AMBR) accounted for approximately 5 million deaths in 2019—a figure projected to reach 10 million by 2050 if no effective interventions are implemented.^3^ In comparison, cancer caused around 10 million deaths in 2022. This number is expected to rise to 87% by 2050.^4,5^ These alarming trends underscore the need to identify novel bioactive molecules during the early stages of drug discovery, characterized by enhanced pharmacodynamic and pharmacokinetic properties, minimal toxicity, and high target selectivity and potency, to overcome resistance mechanisms. Furthermore, identifying alternative biological targets that can be selectively modulated to counteract resistance is promising. In this context, *in silico* techniques may facilitate the rational design of bioactive compounds with optimized pharmacological profiles.^6^ A wide range of computational techniques, including molecular docking, virtual screening, quantitative structure–activity relationships (QSAR), molecular dynamics simulations, and chemoinformatic analyses, are employed atvarious stages of the drug discovery process.^7^

In medicinal chemistry, the use of privileged scaffolds, which are structural frameworks capable of interacting with multiple biological targets, has proven to be an effective strategy. They serve as “chemical navigators” for guiding the development of biologically active molecules.^8^ Among these, 1,5-disubstituted tetrazoles (1,5-DS-TS) have garnered significant attention due to their remarkable biological versatility. As nitrogen-rich heterocycles, these compounds function as bioisosteres of *cis*-amide bonds, often improving metabolic stability and membrane permeability. Consequently, they have been widely explored in the development of antibacterial, anticancer, antifungal, anti-inflammatory, and antiviral agents.^9–12^ Their capacity to modulate key biological pathways makes them attractive candidates in the search for novel therapies to overcome drug resistance.

Given their therapeutic potential, which has been extensively discussed in previous studies,^13–16^ mapping the chemical space of 1,5-DS-TS through chemoinformatics tools can provide medicinal and organic chemists with critical insights to guide decision-making.^17,18^ In the era of big data, it is essential to apply computational methods to identify patterns and uncover hidden relationships within large chemical data sets.^19,20^ Chemoinformatic analyses address these challenges by integrating technology, informatics, and chemical knowledge. Numerous studies have successfully employed chemoinformatics to analyze specific compound libraries, elucidating structure–activity relationships, physicochemical properties, bioactivity profiles, and chemical space exploration.^21–23^ Additionally, chemoinformatic analyses can serve as a starting point to identify bioactive compounds or suggest novel targets through ligand-based interacting patterns.

Building upon our ongoing efforts to design bioactive compounds based on the privileged 1,5-DS-T scaffold, this study presents a chemoinformatic characterization of these heterocycles and a systematic exploration of their chemical space. Our goal is to identify promising candidates for *de novo* synthesis, as well as to suggestpotential therapeutic targets to address drug resistance in cancer and infectious diseases. To the best of our knowledge, no previous study has conducted a comprehensive *in silico* investigation exclusively focused on 1,5-DS-T.

## Materials and Methods

### Data Sets

The data used in this study were retrieved from ChEMBL,^24^ PubChem^25^ and Drug Bank^26^ platforms. In addition, an in-house data set comprising compounds synthesized by the research group of Cortés-García was included (Table 1).^27–33^ The screening procedure applied to each data set was the following:

- PubChem: the initial search for 1,5-DS-T structures yielded 231,268 compounds. Compounds with an associated ChEMBL code were excluded, reducing the set to 199,696. Entries with missing data were then removed (189,102 remaining), followed by the exclusion of molecules lacking 3D structures (183,461). A final filter was applied using AlvaMolecule’s “Check Structures” tool, in which all default structural filters were used (multiple structures, unusual valence atoms, covalent/ionic bonds, total charge, isotopes, charged atoms, nonstandard atoms, aromaticity, and radical atoms). Compounds presenting structural alerts or errors were removed, resulting in a curated data set of 172,852 compounds.
- ChEMBL: compounds containing 1,5-DS-T scaffold were first extracted from the ChEMBL_34 release, yielding 5336 molecules. Compounds without bioactivity data or containing incomplete entries were removed, resulting in an initial data set of 3867 compounds. Scaffold selection in ChEMBL allowed us to filter out compounds for which bioactivity was reported, but no quantitative activity value was provided. Thus, an additional step was required. To obtain IC_50_ values, the 3867 molecules were mapped to a predownloaded collection of 2,097,152 compounds from ChEMBL with reported IC_50_ data, providing 1444 records. To refine the data set, compounds were filtered based on their target name, as this annotation is required for further analysis. Compounds that lacked receptor information, had unspecified receptor types, or listed an organism/cell line (e.g., *Homo sapiens*, *Rattus norvegicus*) as the target annotation were discarded. After this filtering step, 901 compounds remained. The final data set consisted of 718 molecules possessing valid 3D structures and no structural alerts, as determined by AlvaMolecule’s “Check Structures” tool (all default structural filters applied).
- In-house data set: a total of 276 1,5-DS-T have been synthesized in-house. However, only the 170 compounds that have already been published were included in this study.^27–33^

- Data Bank: ten FDA-approved drugs containing the 1,5-DS-T core were included from the DrugBank database. These are cefotetan, cefoperazone, cefmenoxime, cefonicide, cefepime, cefotaxime, latamoxef, cefamandole, cefmetazole, and cefotiam.

**Table 1:** Summary of Data Sets Containing 1,5-DS-T Moiety

	size	
dataset	initial	curated
PubChem	231,268	172,852
ChEMBL	5336	718
In-house	276	170
DrugBank	10	10

### Physicochemical Properties of Therapeutic Relevance

The following molecular properties were computed using the rdkit.Chem.Descriptors.CalcMolDescriptors­() module from RDKit library: molecular weight (MW), number of rotatable bonds (RTB), hydrogen bond acceptors (HBA), hydrogen bond donor (HBD), topological polar surface area (TPSA), heavy atoms count (HA) and the octanol/water coefficient (LogP). The obtained values were normalized using the StandarScaler (). fit_transform () function from the Scikit-learn library prior to conducting the chemical space, complexity, diversity and *k*-means analyses.

### Chemical Space Analysis

The chemical space of the four data sets was analyzed by PCA (Principal Components Analysis) based on seven molecular properties of pharmacological interest: molecular weight (MW), number of rotatable bonds (RTB), hydrogen bond acceptors (HBA), hydrogen bond donor (HBD), topological polar surface area (TPSA), heavy atoms count (HA) and the octanol/water coefficient (LogP). The modules and packages used for these analyses were carried out in Jupyter Notebook, a Python-based programming platform, and the main tools for visualization were matplotlib and seaborn. Key molecular descriptors of pharmacological interest were defined, including molecular weight, hydrogen bond acceptors and donors, logP, rotatable bonds, aromatic rings, and topological polar surface area.

### Diversity Analysis

The normalized ratios of principal moments of inertia, NPR1 and NPR2, were calculated. For this purpose, the RDKit 2024.09.4 package and the rdkit.Chem.rdMolDescriptors module in Jupyter Notebook was used and plotted using the workflow described by Ruiz-Moreno *et al*.^34^

### Scaffold Analysis

Scaffold analysis was performed to identify core molecular frameworks and substitution patterns within the data set. The methodology was adapted from the open-source cheminformatics resource Practical Cheminformatics Tutorials (https://github.com/PatWalters/practical_cheminformatics_tutorials, accessed July 2, 2025), specifically using the workflow implemented in the notebook find_scaffolds.ipynb and its associated module scaffold_finder.py.^35^

### Analysis of Therapeutic Targets of 1,5-DS Tetrazoles from the ChEMBL Data Set

The CSV file of the 1,5-DS-T data set from ChEMBL, containing information on compound IDs, their therapeutic targets, and their bioactivity measurements (IC_50_) was used for this study. IC_50_ values were selected because they are independent of a compound’s specific mechanism of action, making them suitable for comparing activity across different compounds. The analysis was performed using Microsoft Office Excel, version 2021.

### Descriptor-Based Clustering and Associated Targets

Ligand-based target associations were performed using a *k*-means clustering algorithm implemented via the sklearn.cluster module in Python with the calculated physicochemical properties of therapeutic relevance (HBA, RTB, TPSA, HA, MW and LogP). Several approaches exist to estimate the optimal number of clusters (*k*), including methods such as the Elbow, silhouette, and Davies–Bouldin indices. Comparative studies show that while different methods have specific advantages, most perform well on small data sets. Since our data set is small, the Elbow method was selected due to its good performance, low computational cost, and widespread use.^36–38^ The Elbow method (also known as the Jambú elbow criterion) was applied by plotting the within-cluster sum of squares (WCSS) against the number of clusters. Principal Component Analysis (PCA) was subsequently employed to reduce dimensionality and visualize the clustering results in two dimensions.

### Pocket Analysis

For the pocket analysis, CXCR3–CXCL10 and CCR3–CCL24 complexes were modeled. These endogenous ligands were selected for their reported selectivity for their respective receptors and limited cross-reactivity with other chemokine systems. The modeling was carried out using the Boltz-2 foundation model,^39^ following the same protocol described in the Affinity Prediction section of the Materials and Methods. The only modification was that, in the YAML input files, the ligand was defined as a protein. The amino acid sequences of human CXCL10 and CCL24 were retrieved from the UniProt database (accession codes P02778 and O00175, respectively). Likewise, the sequences of the receptors CXCR3 (accession code P49682) and CCR3 (accession code P51677) were obtained from UniProt. These sequences were integrated into the corresponding YAML files together with the modified PDB structures of each receptor (CXCR3: PDB ID 8K2W, chain A; CCR3: PDB ID 7X9Y, chain D). Subsequently, the same modified PDB structures were analyzed using the FPocketWeb^40^ application to identify the binding pockets of each receptor and calculate the corresponding descriptors available within the platform.

### Docking Analysis

Docking studies were conducted using Molegro Virtual Docker 5.0 to calculate the binding energies of the designed compounds. The CXCR3 crystal PDB:8K2W that is cocrystallized with the antagonist AMG-837 was used. The docking process employed MolDock Score [GRID] with a 0.40 Å resolution, MolDock SE, and the following parameters: 10 runs, population size of 150, max iterations 1000, max steps 310, and a neighbor distance of 1.10. The binding site coordinates were *x* = 108.34, *y* = 110.67, *z* = 138.24 and a radius of 10.

### Affinity Prediction

The binding affinities of compounds IH-25, IH-39, IH-114, and AMG-487 were predicted using the Boltz-2 foundation model^39^ with the open-source code available at the official GitHub repository (jwohlwend/boltz). For each compound, a YAML file was generated containing the SMILES representation of the ligand and the amino acid sequence of human CXCR3 (accession code P49682), retrieved from the UniProt database. Additionally, a modified PDB structure of CXCR3 (PDB ID: 8K2W), containing only chain B, was provided as input alongside the corresponding YAML file. The calculations were executed using Git Bash within a Linux-configured environment.

### Literature Analysis

The CSV file of the 1,5-DS-T dataset from ChEMBL, containing information on compound IDs and publication IDs, was used for this study. The articles were manually searched using their publication IDs, and each article was reviewed to determine whether the synthesis had been performed using the Ugi-azide reaction. The analysis was conducted using Microsoft Office Excel, version 2021.

## Results and Discussion

### In-House Library Populates the Chemical Space of ChEMBL and DrugBank Libraries

A collection of four data sets containing the 1,5-disubstituted tetrazole core structure was ensembled and curated. These datasets encompassed: (i) 172,852 compounds retrieved from PubChem, (ii) 718 bioactive molecules with curated IC_50_ values from ChEMBL, (iii) 10 FDA-approved drugs from DrugBank, and (iv) an in-house library of 170 compounds (Figure 1), synthesized at the Cortés-García research group, using Ugi-azide multicomponent reactions. Analyses performed on this collection of 1,5-DS-T are described in the following sections.

**Figure 1:**
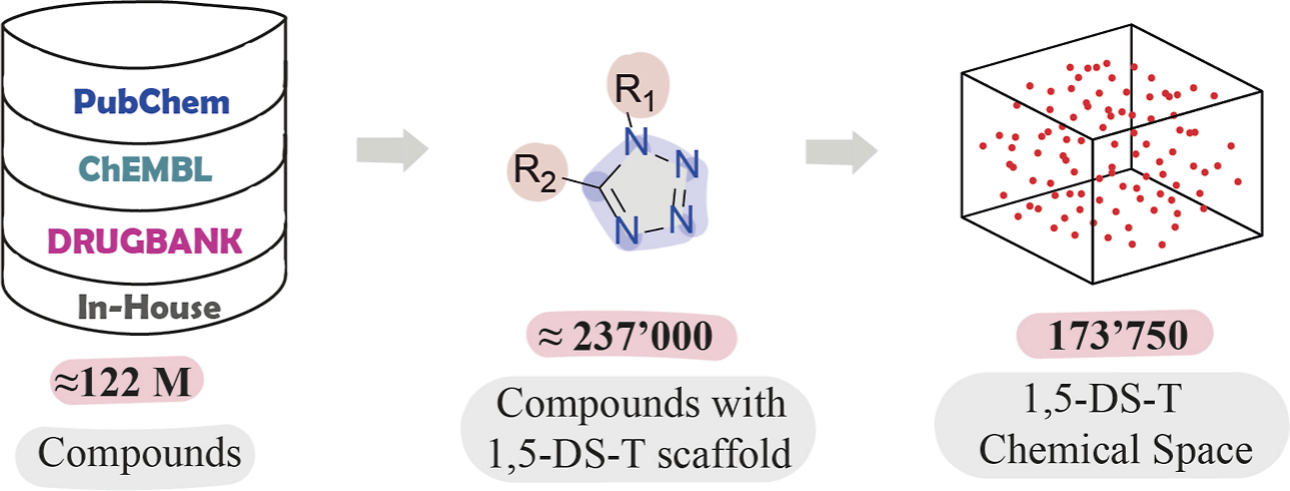
Filtered libraries for constructing the chemical space of 1,5-DS-T derivatives.

To visualize the chemical space of the data sets studied here, principal component analysis (PCA)^41,42^ was performed using seven pharmacologically relevant molecular descriptors (Figure 2a): molecular weight (MW), hydrogen bond acceptors (HBA), hydrogen bond donors (HBD), number of rotatable bonds (RTB), topological polar surface area (TPSA), heavy atom count (HA), and the octanol–water partition coefficient (LogP). The first two principal components accounted for 75.17% of the variance (PC1 = 50.07%, PC2 = 25.10%), as shown in Figure 2b, enabling meaningful 2D visualization of the chemical space. Compounds from PubChem were broadly distributed, in agreement with significant chemical diversity. Interestingly, the FDA-approved drugs analyzed occupied a distinct region characterized by larger molecular weight (>450 Da), lower lipophilicity (LogP <1), and increased polarity (TPSA >200 Å^2^) and a count of heavy atoms above 30. The distribution of data points shows that the compounds in the in-house data set occupy a region of the chemical space largely overlapped by the ChEMBL data set. This overlap suggests that the in-house compounds share physicochemical properties with 1,5-DS-T reported in ChEMBL. This profile highlights the potential of 1,5-DS-T compounds in the in-house collection as a privileged scaffold in early stage drug discovery. Given the need to overcome drug resistance—particularly in pathogens and cancer types that have developed tolerance to conventional therapies—the diversity and drug-likeness of the 1,5-DS-T chemical space may offer promising starting points for the development of novel agents capable of circumventing resistance mechanisms.^43^

**Figure 2:**
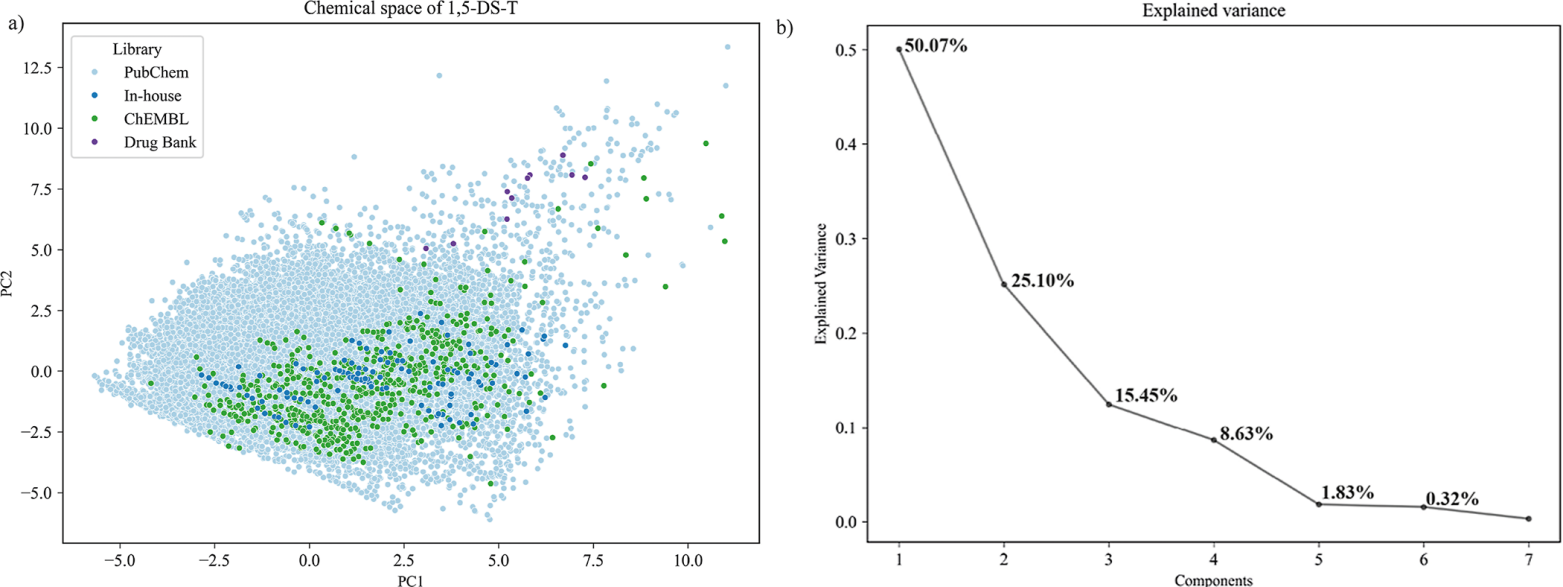
(a) Visualization of the chemical space of 1,5-DS-T by Principal component analysis. (b) Percentage of variance explained by components PC1 and PC2.

To further characterize drug-likeness, key physicochemical properties were analyzed across all libraries. Most 1,5-DS-T compounds had molecular weights between 200–500 g/mol, around six HBA, one HBD, TPSA values of 80–90 Å^2^, approximately 20–25 heavy atoms, and LogP values between 1–2. These characteristics are largely consistent with Lipinski’s Rule of Five,^44^ indicating potential oral bioavailability. Overall, 96.81% of the compounds in the data set comply with Lipinski’s criteria, including 167,442 compounds from PubChem, 642 from ChEMBL, 2 from DrugBank, and 103 from the in-house library. In contrast, a smaller fraction of the data set (0.71%, corresponding to 1233 compounds from PubChem) satisfies the Rule of Three criteria, a rule which is typically associated with fragment-based drug discovery (FBDD),^45^ suggesting that a subset of 1,5-DS-tetrazole compounds may be suitable for fragment-like as well as lead-like development strategies.

### Complexity and Diversity Analysis

To assess three-dimensional molecular shape diversity, normalized principal moments of inertia (PMI) were computed and visualized (Figure 3a).^46^ PMI plots provide a means to visualize, compare, and quantify the molecular complexity of a set of compounds based on their three-dimensional molecular shape and enable the classification of molecules into rod-like, disc-like, or spherical shapes. The majority of compounds are located within the rod-disc region, indicating a prevalence of linear and planar structures dominated by sp^2^ and sp hybridization. These structural characteristics are associated with improved membrane permeability and π–π stacking interactions, which facilitate binding to flat or aromatic surfaces in biological targets such as enzyme active sites and receptor binding pockets.^47^ Interestingly, the in-house library mirrored the ChEMBL data set in PMI distribution, a relevant feature for molecular recognition and target binding, reinforcing its potential biological relevance. In contrast, the PubChem library exhibited a broader shape diversity, molecules in PubChem span the entire rod-to-disc continuum and even approach the sphere-like region, suggesting a higher topological complexity and flexibility in this data set. While DrugBank compounds were confined to more compact, rod-like and disc-like areas—a likely outcome of clinical optimization processes. Compounds with higher three-dimensionality tend to exhibit a greater likelihood of success in clinical stages, reduced promiscuity, and a broader spectrum of biological activity.^48,49^

**Figure 3:**
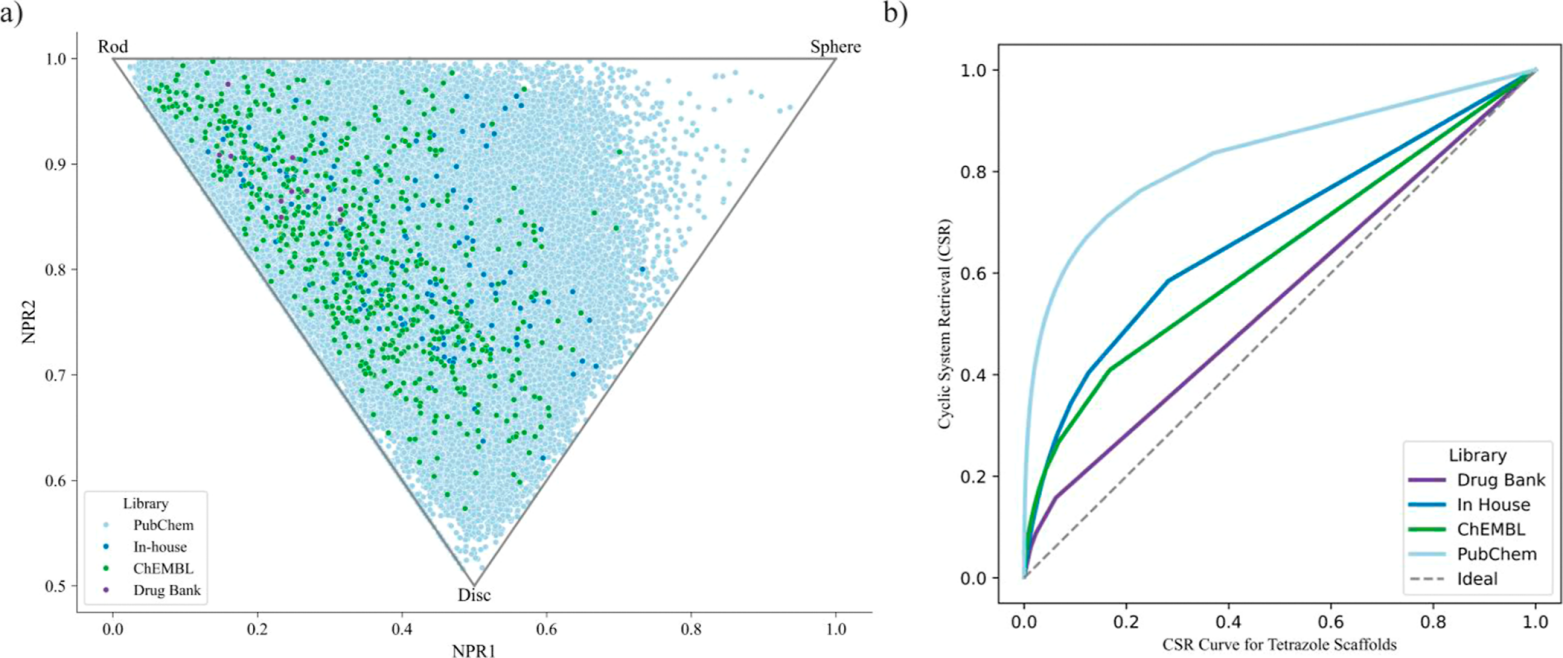
(a) Analysis of the principal moments of inertia (PMI) of ChEMBL, PubChem, Drug Bank and in-house data sets. (b) Cyclic system retrieval (CSR) curves for the four libraries.

Scaffold diversity was then assessed using Cyclic system retrieval (CSR) curves, also known as cumulative scaffold frequency plots (CSFPs), which are generated by plotting the cumulative percentage of molecules against the cumulative frequency of unique scaffolds.^50,51^ The closer the curve is to the diagonal (ideal line), the greater the scaffold diversity, indicating a more uniform distribution of distinct scaffolds across the data set. As shown in Figure 3b, the DrugBank data set showed the highest scaffold diversity, with nearly every molecule possessing a unique scaffold, despite its limited size. Consistent with Bajorath and Hu, that reported an analysis of 1241 approved drugs with 700 scaffolds—of which 79% were unique to a single approved drug, while only 39% represented multiple approved drugs.^52^ The ChEMBL library also exhibited considerable diversity, while the in-house and PubChem libraries display lower scaffold diversity as their CSR curves deviate further from the diagonal. The diversity of substitution patterns can be increased by molecular hybridization strategies through the synthesis of polyheterocyclic systems via Ugi-azide multicomponent reactions, thereby enriching the chemical space of these tetrazoles for the more efficient development of new molecules containing the 1,5-DS-T scaffold. To explore this proposal, the substitution patterns within the 1,5-DS-T chemical space were analyzed from the scaffold analysis previously performed. The scaffold workflow applies the FragmentMol function from the matched molecular pair analysis (MMPA) module in RDKit. A matched molecular pair is defined as pairs of compounds that only differ by a chemical change at a single site while sharing a common molecular framework.^53^ Since all the molecules contain the 1,5-DS-T core, the most frequent scaffold reflects the predominant substitution pattern of the chemical space.

As shown in Figure 4, the 25 most common scaffolds exhibit an apparent prevalence of phenyl rings at either position 1 or 5, as well as aniline derivatives, primarily at position 5. Additionally, short-chain alkyl groups such as methyl, ethyl, and propyl, along with aminoalkyl substituents, are frequently observed. Interestingly, scaffold 13 is the only example featuring a quinolinone group, indicating that polyheterocyclic systems are scarcely explored in the current data set of 1,5-disubstituted tetrazoles, which suggests a promising opportunity to expand structural diversity by incorporating more complex heterocyclic motifs. These findings underscore the importance of moving beyond the conventional substitution motifs—such as phenyl groups and small alkyl chains—to fully harness the chemical and pharmacological potential of the 1,5-DS-T scaffold. Expanding the substitution landscape to include a broader range of polar, heterocyclic, or three-dimensionally complex functional groups could significantly increase scaffold diversity and improve the likelihood of identifying novel, drug-like compounds, particularly for addressing urgent challenges such as drug resistance.

**Figure 4:**
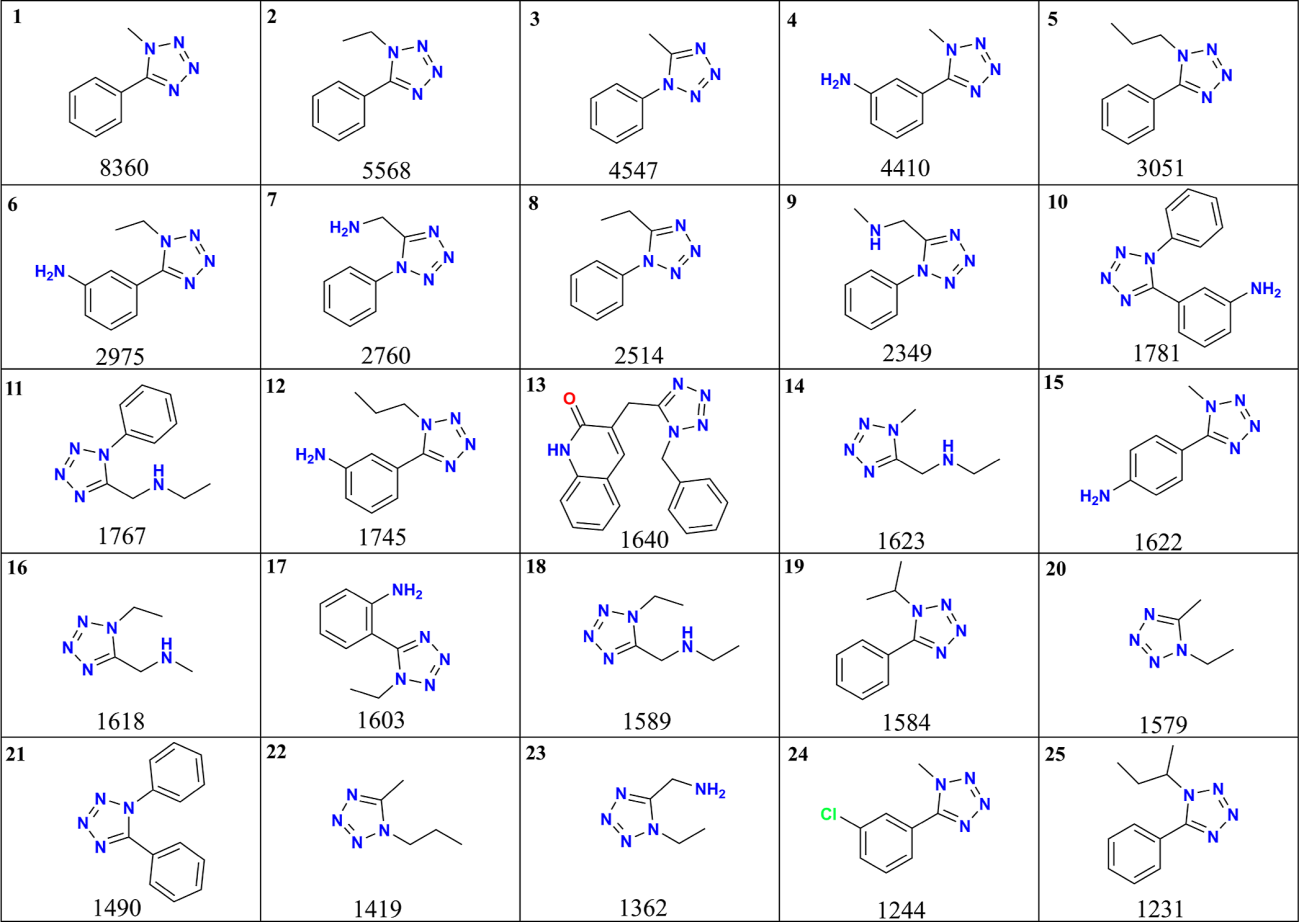
Most frequent scaffolds in the 1,5-DS-T chemical space.

### Bioactivity and Therapeutic Targets Analysis

To explore the biological relevance of 1,5-DS-T, we examined therapeutic targets associated with 718 compounds from the curated ChEMBL data set, each annotated with a single IC_50_ bioactivity measurement. As shown in Figure 5a, the majority of the bioactive compounds were target enzymes, followed by membrane receptors, ion channels, and transcription factors. Notably, a significant number of compounds also act on transporters and epigenetic regulators, such as kinases and histone-modifying enzymes, which are increasingly being recognized as druggable targets in various disease contexts. These findings support the notion that the 1,5-DS-T moiety is a privileged structure capable of multitargeted activity and may have improved efficacy by hitting more than one target synergistically and thus limiting drug resistance, that arises from mutations, efflux mechanisms, or compensatory signaling pathways.^54^ Furthermore, 1,5-DS-T core structure allows for the synthesis of structurally diverse molecules (*vide infra*) that can be explored to overcome resistance in infectious diseases and cancer.

**Figure 5:**
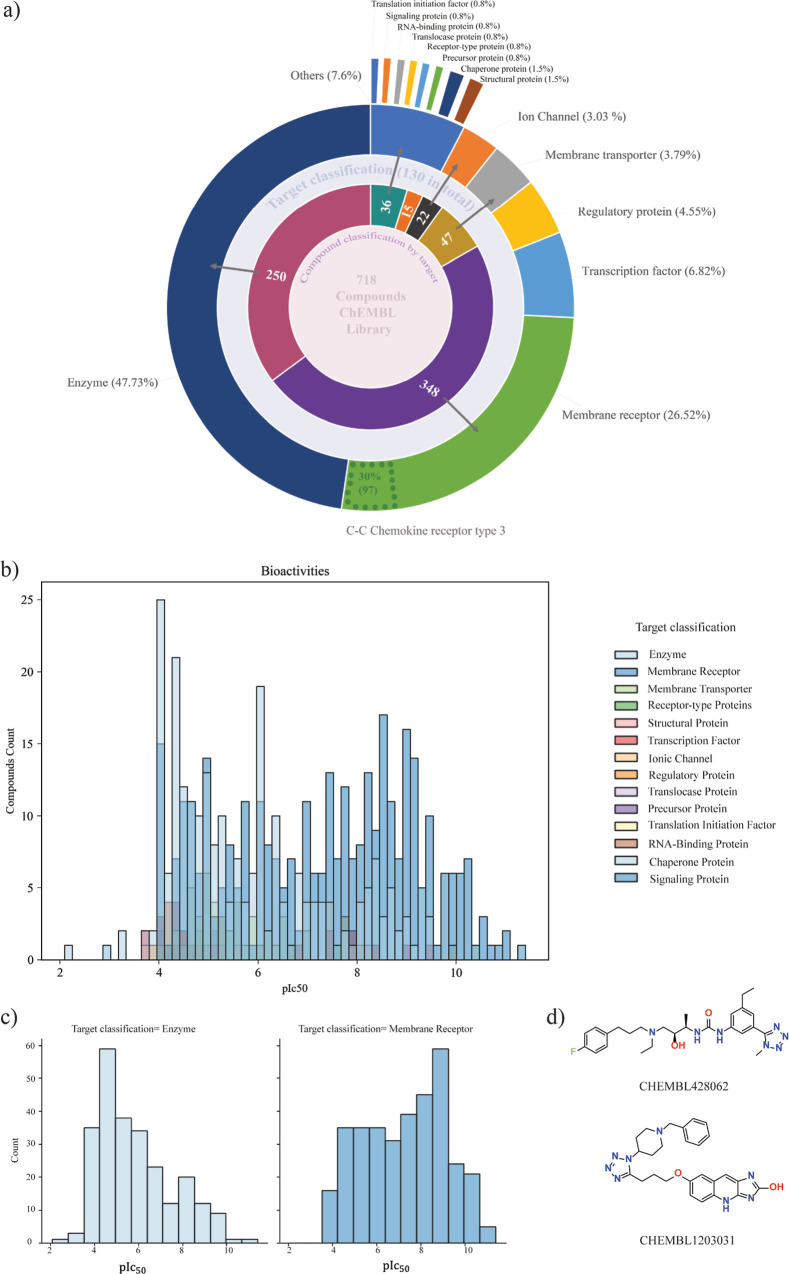
(a) Number of therapeutic targets associated with IC_50_ measurements of the ChEMBL data set. (b) pIC_50_ activities of the ChEMBL library. (c) Highest pIC_50_ values. (d) Compounds with the highest pIC_50_ values.

The distribution of pIC_50_ values^55^ by therapeutic target class, ranges from 2 to 11.4 as shown in Figure 5b the higher the pIC_50_ value the higher the potency. The most potent compounds primarily target enzymes and membrane receptors, while compounds with lower pIC_50_ values are more frequently associated also with enzymes and translational initiation factors. Notably, two compounds displayed exceptional potency, each with a pIC_50_ of 11.4: CHEMBL1203031, an antagonist of the C–C chemokine receptor type 3 (CCR3), which is implicated in asthma therapy, and CHEMBL428062, a synthetic inhibitor of HMG-CoA reductase designed to lower blood cholesterol levels (Figure 5c,d).

Collectively, these findings highlight the ability of 1,5-DS-T to achieve nanomolar-level potency against pharmacologically important targets and to achieve high target-specific potency, particularly those intended to complex pathologies such as antimicrobial resistance and chemoresistant cancers.

### Ligand-Based Target Prediction for In-House Compounds

As a first step toward identifying a potential biological target, molecules from the in-house tetrazole collection were subjected to *k*-means clustering alongside tetrazoles reported in ChEMBL. The visualization of the chemical space of these two subsets, based on the therapeutic properties, is shown in Figure 6a. The variance explained with the first two principal components is 78.38% (Figure S1).

**Figure 6:**
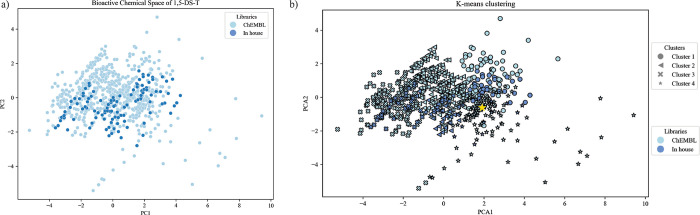
(a) 2D visualization of the chemical space of 1,5-DS-T comprising the ChEMBL library (ii) and the in-house library (ix) using PCA. (b) *k*-means clustering analysis of the chemical space of T-1,5-DS, the star shows the coordinates of the control compound.

Unsupervised clustering was then applied using the *k*-means algorithm. The optimal number of clusters (*k* = 4) was determined using the elbow method, a standard heuristic method, based on the minimization of intracluster variance. Importantly, all the properties (coordinates) were used for the clustering. Once *k* is set, the algorithm assigns initial random centroid coordinates in the feature space. Then, it calculates the Euclidean distance between each data point and the nearest centroid, grouping points accordingly and updating centroids iteratively until convergence is achieved.^56,57^ Each compound was assigned to one of four clusters. The four clusters identified are shown in Figure 6b: Cluster 1 (186 compounds), Cluster 2 (210 compounds), Cluster 3 (208 compounds), and Cluster 4 (284 compounds).

The known bioactive compound CHEMBL428062, identified previously as the most potent ligand (pIC_50_ = 11.4), was used as a reference point to guide the clustering process. Compound CHEMBL428062 is located in Cluster 4 and targets the C–C chemokine receptor type 3 (CCR3). Interestingly, in the ChEMBL library, 93 compounds have reported IC_50_ bioactivity for CCR3. Of these, 72 are grouped in Cluster 4, suggesting that the *k*-means algorithm is effectively grouping compounds based on their bioactivity. Consistent with previous reports, *k*-means clustering has proven to be a valuable approach for discriminating between active and inactive compounds within bioactivity-driven datasets and query compounds.^58–61^ Building upon this, 59 in-house compounds are located in Cluster 4—the second most populated cluster with in-house molecules—and, based on the similarity principle,^62–64^ these compounds may exhibit activity against CCR3. Predictive models of CCR3 activity are warranted. To note, a three-dimensional structure with a cocrystallized ligand of CCR3 is not yet available, and it is primarily associated with allergic and inflammatory diseases.^65,66^ Given the research group’s interest in cancer, and considering that the chemokine receptor family comprises nearly 48 members, an analysis was conducted to determine whether other chemokine receptors exhibit similarity to CCR3 and could therefore represent alternative molecular targets for the 1,5-DS-T library.

Chemokine receptors belong to the class A of G protein-coupled receptor (GPCR) superfamily and are grouped into five subfamilies—CC, CXC, XC, CX_3_C and ACKRs—distinguished by the relative positioning of two conserved cysteine residues near the N-terminus. In fact, other chemokine receptors are also promising, with relevance in cancer and drug resistance.^67,68^ For this reason, the accession code (P51677) of CCR3 was used in the BLAST tool to identify other chemokine receptor with higher relevance to our cancer-related research interests. As a result, the CXC subfamily emerged within the top 20 with highest sequence identity (Figure S2), specifically the chemokine CXCR3. Since the top-ranked receptors belonged to the CC subfamily, we performed a complementary literature search in PubMed using the terms “CC chemokine receptors cancer” and “CXC chemokine receptors cancer” to asses which chemokine receptor subfamily has been more extensively investigated in the context of cancer. The research retrieved 4′786 publications for CC receptors and 8′225 for CXC receptors, indicating a stronger association of the CXC subfamily with cancer-related research. Based on this observation, we focused on CXC receptors. Among them, CXCR3 was selected because it exhibited the highest sequence similarity with CCR3. Additionally, CXCR3 chemokines (CXCL11, CXCL10, and CXCL9) can bind to CCR3 and inhibit its signaling through competition with its endogenous ligands. Conversely, CXCR3 is also capable of binding and sequestering ligands of CCR3.^69–71^ These observations suggest that the binding pockets of both receptors share a certain degree of geometrical and physicochemical properties.

To elucidate the shared properties of both binding pockets, they were analyzed using the FPocket Web application. Since only one crystal structure of CCR3 has been reported and lacks its endogenous ligands, Boltz-2 was used to model the receptor–ligand complex to identify the binding surface (see Materials and Methods for details). The results are presented in Table S2 and Figure S3. For CXCR3, the highest-ranked pocket, located at the same site as the binding region in the Boltz-2 complex, has a total volume of 1765 Å^3^, an Apolar surface area of 65.36 Å^2^, a hydrophobicity score of 25.85, and a polarity score of 21, while no polar surface area was detected. In contrast, the analysis of CCR3 identified four pockets that together constitute the binding region, consistent with the binding interface observed in the Boltz-2 complex model. The combined volume of these pockets is 1451 Å^3^, and no polar surface area was detected. Their hydrophobicity scores range from 45 to 62, while their polarity scores range between 4 and 6. These results indicate that both binding pockets exhibit a mixed hydrophilic–hydrophobic character, with CCR3 being more hydrophobic than CXCR3. Additionally, the CXCR3 binding pocket appears as a more continuous cavity, whereas the CCR3 binding region is more fragmented, composed of multiple subpockets. Differences in polarity and hydrophobicity scores suggest that these features contribute to receptor selectivity toward their respective ligands. At the same time, the shared mixed physicochemical properties provide a rationale for their ability to accommodate overlapping ligands. Nevertheless, a crystallized structure of CCR3 in complex with a ligand is required to validate these predictions and to enable a more detailed characterization of its binding pocket.

As mentioned previously, cancer therapies increasingly face drug resistance across multiple cancer types. This resistance arises through several mechanisms, including efflux pump overexpression, enhanced DNA repair, evasion of apoptosis, and the influence of the tumor microenvironment (TME), among others.^72^ The TME refers to the extracellular milieu in which tumor cells reside and comprises cancer cells, cancer-associated fibroblasts, immune cells, the extracellular matrix, and the vascular network. It has been demonstrated that all components of the TME interact to actively promote cancer progression and therapeutic resistance.^73–76^ Therefore, recent studies have also shown that, in several solid tumors, CXCR3 and its chemokines play an essential role within the TME, exerting either tumor-promoting or tumor-inhibiting effects depending on the cancer type.^77,78^ For example, activation of the CXCL9/CXCR3 axis has shown to induce epithelial–mesenchymal transition (EMT), cytoskeletal reorganization, and enhanced invasion and metastasis via AKT pathway activation in tongue squamous cell carcinoma and other solid tumors, thereby contributing to a more aggressive and drug-refractory phenotype.^79^ Similarly, CXCR3 overexpression confers sorafenib resistance in hepatocellular carcinoma models, whereas CXCR3 blockade or silencing restores sensitivity by reversing CXCR3-dependent metabolic adaptations.^80^ Beyond intrinsic tumor cell resistance, CXCR3 also plays a role in immune escape and response to immunotherapy: CXCR3^+^ regulatory T cells have been implicated in intratumoral immune suppression, and combined modulation of the CXCR3 axis—for example, CXCR3 antagonism together with a STING agonist—has been reported to overcome anti-PD-L1 resistance in lung adenocarcinoma models.^81^

Overall, CXCR3 has emerged as a biologically and pharmacologically relevant target, given its strong association with chemoresistance, tumor progression, metastasis, and immune modulation.^82–85^ In addition to the growing mechanistic evidence linking CXCR3 to therapy resistance, recent reviews have emphasized that this receptor is also a pharmacologically actionable target for drug discovery, with multiple small-molecule antagonists already developed and some, such as AMG-487 and ACT-777991, having progressed to clinical evaluation.^86^ Although no CXCR3-targeting agent has yet received FDA approval, the accumulated preclinical and early clinical evidence support CXCR3 as a credible and underexplored therapeutic target in drug-resistant cancers.^87,88^ This makes CXCR3 an attractive target for the discovery of structurally novel 1,5-DS-T-based ligands with selective affinity and therapeutic potential. Additionally, these findings are consistent with the work of Ertl *et al.*, who performed a chemoinformatic analysis of ring systems and reported a preference of tetrazole ring for GPCRs as a target class. Such observations further support the relevance of exploring 1,5-DS-T compounds within this pharmacological space.^89^ Such efforts could aid the development of new anticancer agents capable of addressing chemoresistant tumors.

### Molecular Docking of In-House Compounds with CXCR3 and Affinity Prediction

Following the identification of CXCR3 as a promising therapeutic target, molecular docking studies were conducted to evaluate the binding potential of 170 compounds from the in-house 1,5-DS-T library (Figures S5 and S6). These simulations were carried out using the crystal structure of the human CXCR3 receptor (PDB ID: 8K2W), cocrystallized with the known antagonist AMG-487 (Figure 7a). This structural model served as the basis for evaluating the binding potential and interaction profiles of the 1,5-DS-T derivatives within the orthosteric site of the receptor.

**Figure 7:**
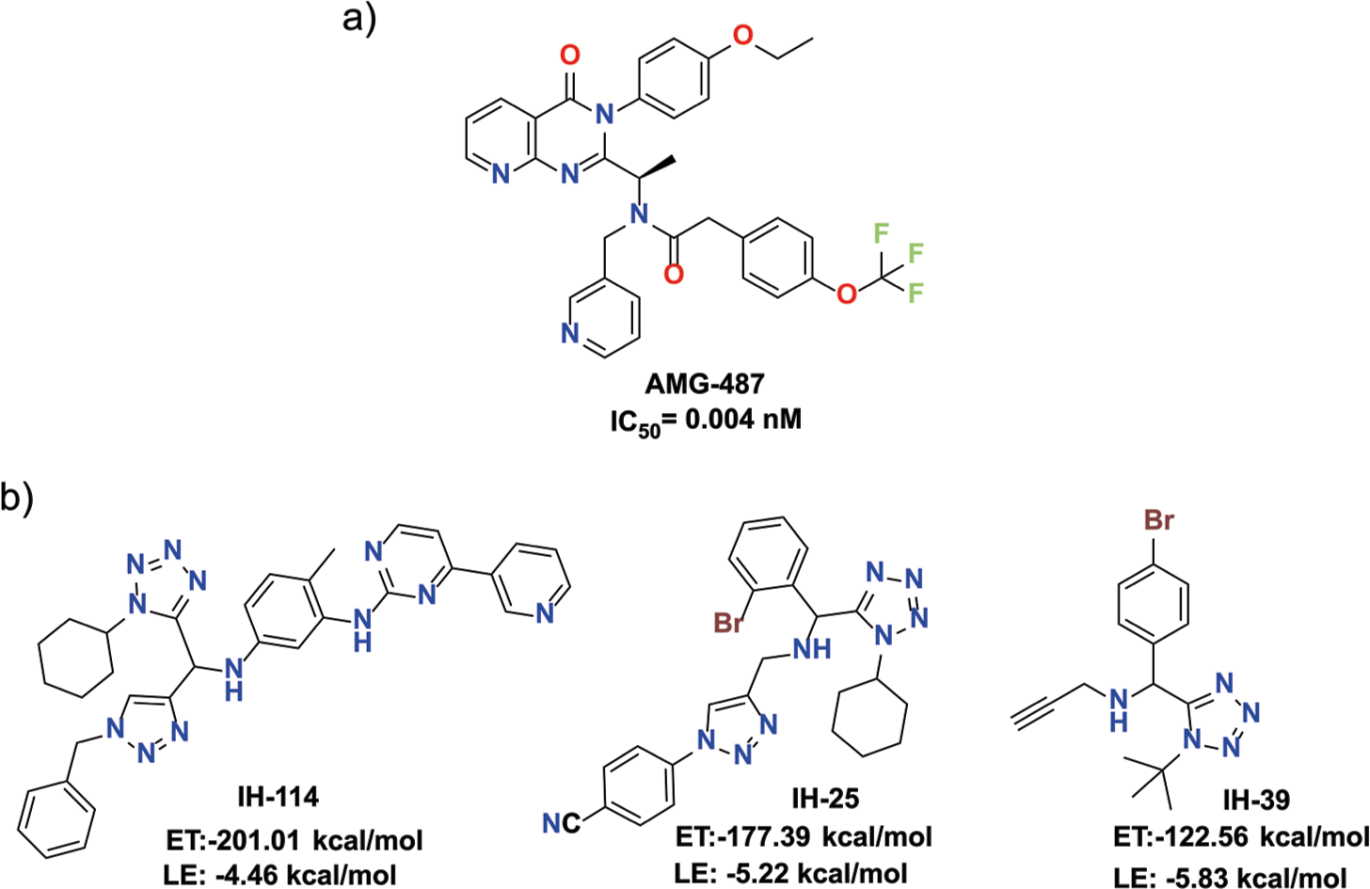
(a) CXCR3 antagonists with nM activity that have reached the clinical phase. (b) In-house compounds that showed the lowest ligand efficiency (LE), the lowest binding energy or a combination of both metrics.

The cocrystallized ligand, AMG-487, was redocked with a root-mean-square deviation (RMSD) of 1.45 Å (accepted threshold for docking validation ≤2.0 Å), a docking score of −167.80 kcal/mol, and a ligand efficiency of −3.81. kcal/mol. Key interactions between AMG-487 and the CXCR3 binding pocket included hydrogen bonds with Cys203, Tyr316, Tyr69, Ser312, Tyr279, and Lys308, as well as π–π interactions involving Trp109, Leu63, and Leu59. Notably, interactions with Trp109 and Tyr60—highlighted by Jiao *et al*. as critical for molecular inhibition—were successfully reproduced. It has also been reported that the orthosteric site of CXCR3 consists of a cluster of aromatic residues, including Tyr60, Trp109, Phe131, Phe135, Tyr205, Trp268, Tyr271, and Tyr308.^90^ These residues are important for the binding and activation of CXCR3 by its endogenous chemokines, CXCL9, CXCL10, and CXCL11. Importantly, orthosteric antagonists also engage this same binding region; however, unlike the natural chemokines, they occupy the site without triggering receptor activation. Therefore, effective antagonists must compete with these natural ligands by targeting the same site with high specificity and selectivity.^91^

The docking results are summarized in the Supporting Information (Table S1), with particular emphasis on the ten compounds exhibiting the lowest binding energies combined with a low ligand efficiency. Among these, three standout candidates were selected for detailed analysis and are shown in Figure 7b. The selection criteria focused on two key parameters: total binding energy (E-Total) and ligand efficiency (LE). Compound **IH-114** demonstrated the most favorable E-Total (−201.01 kcal/mol), suggesting a strong overall binding affinity. **IH-25** exhibited a balance of favorable binding energy and LE (−177.39 kcal/mol and −5.22 kcal/mol, respectively), making it a compelling candidate in terms of both potency and efficiency. **IH-39**, while displaying a less favorable E-Total (−122.56 kcal/mol), had the highest LE value (−5.83 kcal/mol), indicating high binding efficiency relative to its molecular size. These three compounds were subjected to further analysis to examine their binding modes and specific molecular interactions within the CXCR3 orthosteric site. In addition, PAINS analysis performed with SwissADME indicated that **IH-25**, **IH-39**, and **IH-114** are free of PAINS alerts, further supporting their consideration for posterior analysis.^92^

Among the in-house compounds located in the CXCR3-related region of the chemical space, **IH-114** was selected as the most promising candidate for further analysis. The evidence supporting this prioritization is described below through docking, Boltz-2 predictions, and binding-mode inspection.

To identify the most promising compound, the first selection criterion was based on a visual inspection of molecular interactions. Fischer *et al.* have emphasized that visual inspection is a critical step following molecular docking, especially for validating docking results and prioritizing ligands. Important aspects to evaluate include key interactions with functionally relevant residues within the binding pocket, the degree of similarity between the binding pose of candidate ligands and that of the cocrystallized reference compound, and the presence of multiple stabilizing hydrogen bonds, which are often indicative of strong and specific binding.^93^

The interaction analysis of **IH-114** revealed that this compound closely mimics key binding features of the CXCR3–AMG-487 complex (Figure 8). Specifically, **IH-114** engages in π–π stacking interactions with Tyr316 and Phe131, and forms hydrogen bonds with His202, Ser309, Tyr279, and Lys308. Furthermore, it establishes π–cation interactions with Lys308 and π–anion interactions with Asp112, reinforcing the strength and specificity of its binding. For an improved visualization of its interactions within the binding site, additional images can be found in Supporting Information (Figure S4). Given the high number of favorable interactions, its superior total binding energy (E-total), and a ligand efficiency (LE) that surpasses that of AMG-487, **IH-114** was selected as the most promising computational hit from this docking campaign.

**Figure 8:**
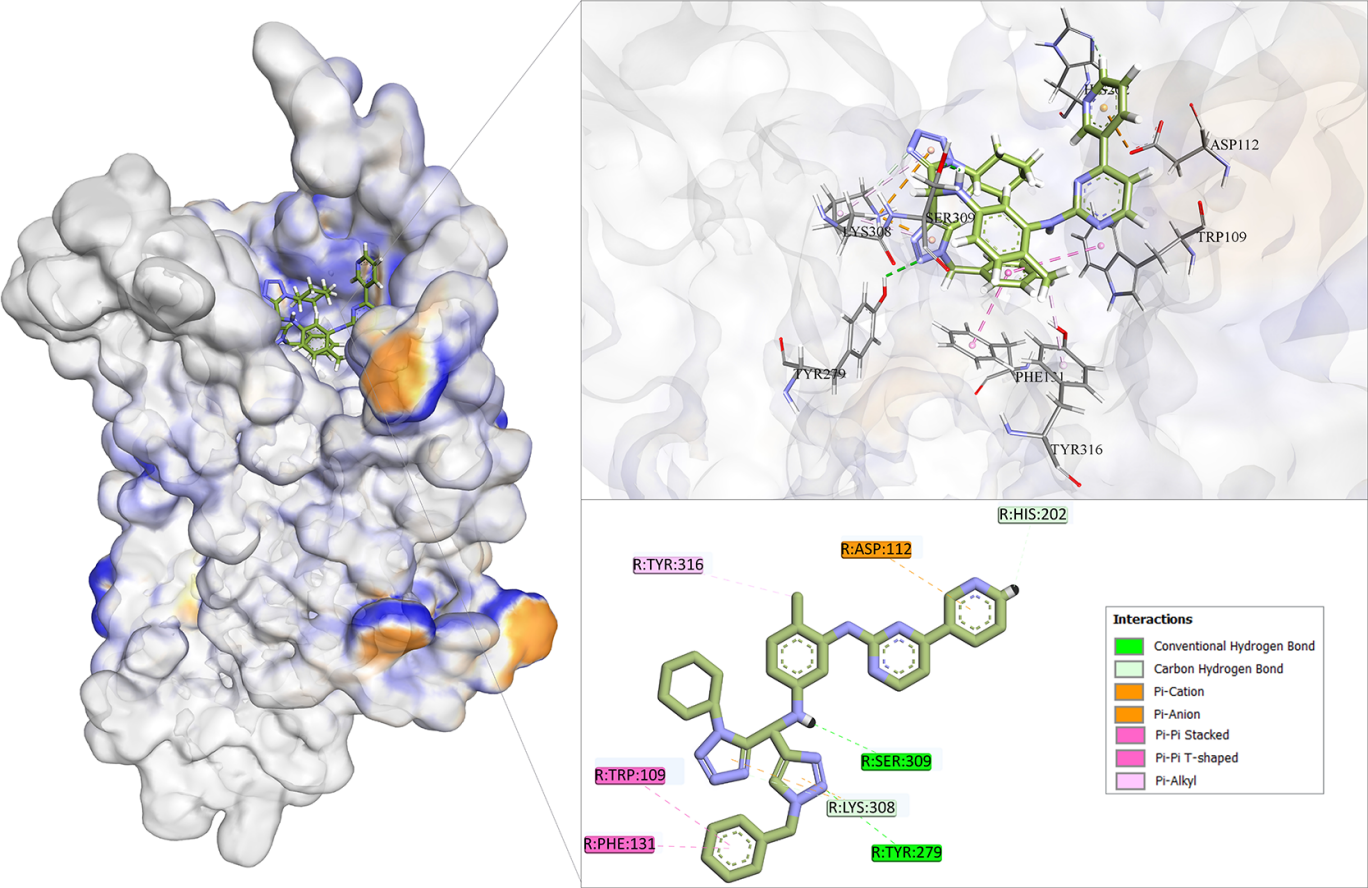
Interactions between compound **IH-114** and CXCR3 orthosteric site.

Even though docking campaigns have been widely used for virtual screening, classical physics-based docking methods still present inherent limitations (see references for further details).^94,95^ In particular, their ability to reliably distinguish true binders from decoys (false positives) remains limited. This shortcoming is mainly attributed to approximations in scoring functions, which often neglect or oversimplify entropic and solvation effects, as well as to the incomplete treatment of receptor–ligand flexibility.^96,97^ Consequently, docking results should be postprocessed to validate and reinforce the prioritization of potential hits identified during the screening campaign.^98^ One way to address this issue is through the use of artificial intelligence models. Recently, diffusion models have attracted considerable attention for their high accuracy in predicting receptor–ligand complexes and binding affinity.^99–101^ Boltz-2 is a recently developed biomolecular foundation model capable of predicting receptor–ligand structures and binding affinities.^39^ To strengthen the evidence supporting the docking results, the open-source Boltz-2 implementation was employed to re-evaluate the binding potential of **IH-114, AMG-487**, **IH-25**, and **IH-39** were analyzed in parallel, and the corresponding results are summarized in Table S3. All four complexes (CXCR3-ligand) yielded confidence scores above 0.78, indicating consistent model predictions. Boltz-2 correctly prioritized the known antagonist AMG-487 as the strongest binder, with a predicted affinity score of −1.12. This value is in qualitative agreement with the high potency reported for AMG-487 in ChEMBL (0.005–30 μM), supporting the model’s ability to recognize a validated CXCR3 binder. **IH-114** displayed an affinity score of −0.43, consistent with moderate-to-strong binding and supporting its selection as the most promising in-house hit. By comparison, **IH-25** showed a weaker predicted affinity of 0.84, whereas **IH-39** showed a score of 1.71, indicating that this compound behaves as a decoy rather than a binder. Overall, the Boltz-2 predictions were broadly consistent with the docking-based prioritization of **IH-114** and **IH-25** over **IH-39**, although the rank order did not fully match that obtained with docking, particularly in the case of AMG-487. This difference further supports the use of Boltz-2 as a complementary postdocking validation step rather than as a direct substitute for docking scores.

The affinity predicted for **IH-114** may be rationalized, at least in part, by its structural architecture, **IH-114** contains a phenylaminopyrimidine (PAP) scaffold together with two additional privileged heterocyclic motifs: a 1,5-disubstituted tetrazole and a 1,4-disubstituted 1,2,3-triazole. These ring systems are widely recognized in medicinal chemistry due to their favorable physicochemical properties, bioisosteric behavior, and frequent incorporation into bioactive molecules, including compounds with reported anticancer activity.^15,43,102,103^ PAP frameworks, for example, constitute the central pharmacophore of several approved anticancer agents.^104^ In contrast, 1,5-disubstituted tetrazole and 1,2,3-triazole rings are found in inhibitors targeting diverse oncogenic targets, including kinases, topoisomerases, tubulin, and components of the PI3K/AKT pathway.^43,105^ Their electron-rich aromatic character supports strong hydrogen bonding, π–π stacking, and electrostatic interactions, enabling binding across structurally diverse protein environments.^106,107^

In the context of **IH-114**, the coexistence of PAP, 1,5-disubstituted tetrazole, and 1,2,3-triazole motifs underscores its structural robustness and ligand-like character. Importantly, this observation is intended to emphasize the chemical suitability of **IH-114** as a potential CXCR3 modulator and not to imply any mechanistic similarity to kinase-targeting PAP derivatives. However, as with any privileged scaffold, particularly those also found in kinase inhibitors, the potential risk of off-target kinase interactions and associated selectivity liabilities should be considered and will require experimental evaluation in future studies. This structural perspective provides a rationale for considering **IH-114** as a promising scaffold for further optimization toward CXCR3 antagonism, particularly in the broader context of identifying new chemokine-receptor modulators relevant to drug-resistant cancers.

### Bioactive 1,5-DS-T Synthesized by the Ugi-Azide Multicomponent Reaction

Lastly, this section aims to explore which compounds within the ChEMBL1,5-DS-T database have been synthesized via the Ugi-azide multicomponent reaction (UA-MCR), which is a powerful synthetic tool for generating highly functionalized libraries of 1,5-disubstituted tetrazoles with broad diversity and structural complexity by varying three points of diversity: primary or secondary amines, aldehydes or ketones, and isocyanides.^108^ By combining these components in a combinatorial manner, the reaction provides rapid, straightforward access to a wide range of 1,5-DS tetrazole derivatives from commercially available or readily accessible starting materials. UA-MCR is among the most practical synthetic methods for the direct, single-step preparation of 1,5-disubstituted tetrazoles under mild conditions and with high operational simplicity.^109,110^ Another significant advantage of the Ugi–azide reaction is its capacity to serve as a synthetic platform for the construction of hybrid pharmacophoric compounds via postcondensation transformations, many of which can be carried out in a one-pot manner, a synthetic strategy that our research group has actively explored.^27–33^ Moreover, the high diversity obtained from the UA-MCR enables exploration and population of the chemical space, proving an efficient strategy for generating structurally diverse analogues with potentially optimized biological profiles, reinforcing its value as a starting point for medicinal chemistry. Given its versatility and efficiency in generating molecular diversity, it is particularly relevant to assess how extensively this methodology has been employed in the synthesis of bioactive 1,5-DS-T derivatives reported in public databases. From the total 718 bioactive 1,5-DS-T compounds identified in the ChEMBL chemolibrary, 135 are linked to original research articles. However, only 88 of these compounds were synthesized through the Ugi-azide MCR, as documented in just nine publications Figure 9.

**Figure 9:**
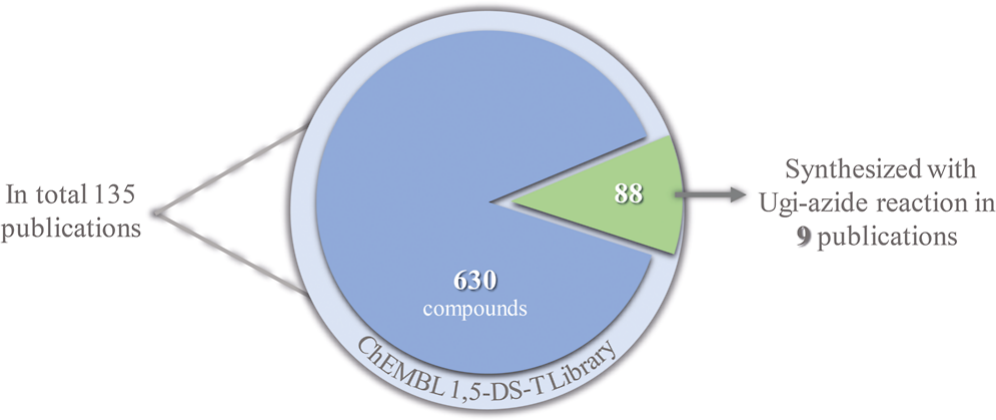
Publications linked to the ChEMBL library of 1,5-DS-T that carried out a Ugi-azide synthesis.

This finding underscores the underrepresentation of the Ugi-azide reaction as a strategy for accessing bioactive tetrazoles, despite its versatility and efficiency. In contrast, most reported 1,5-DS-T compounds have been synthesized through conventional methods such as the [3 + 2] cycloaddition of azides and nitriles or the dehydration of amides using reagents like POCl_3_ and PCl_5_.

Given the limited number of bioactive compounds currently derived from the Ugi-azide multicomponent reactions, there remains a substantial number of molecules with this scaffold to be explored. Expanding this scaffold through Ugi-azide-based diversity-oriented synthesis not only aligns with our group’s research focus but also offers significant opportunities for discovering novel 1,5-disubstituted-tetrazole-based candidates with therapeutic potential. This update reinforces the importance of further exploring this reaction for the efficient generation of diverse and pharmacologically relevant 1,5-DS-T scaffolds.

## Conclusion

This work represents an effort to systematically analyze 1,5-DS-T from diverse compound libraries, with a particular focus on molecules synthesized in-house. The chemical space analysis of 1,5-DS-T revealed that this space can still be significantly expanded through the introduction of new substitution patterns, thereby increasing the structural diversity and complexity of this class of compounds. Additionally, as revealed by the chemical space exploration, 1,5-DS-T are associated with a wide range of bioactivities across various targets and diseases. Increasing their structural diversity could enable the identification of new molecular targets or enhance the activity against already validated ones.

Building on these results, ligand-based target prediction and *k*-means clustering identified CXCR3 as a plausible target for the in-house collection, given its role in cancer drug resistance. Structure-based analyses then prioritized **IH-114** as the most promising computational hit. Docking revealed a favorable interaction profile at the CXCR3 orthosteric site, while Boltz-2 provided complementary AI-based support for its proposed binding. Although these findings do not constitute experimental validation, they support **IH-114** as a structurally robust and pharmacologically relevant starting point for further optimization. In this context, **IH-114** may serve as a starting point for the design of new analogues that retain the key pharmacophoric features of its phenylaminopyrimidine, 1,5-DS-T, and 1,2,3-triazole motifs, with the aim of improving pharmacodynamic and pharmacokinetic properties and guiding subsequent *in vitro* evaluation against CXCR3.

Finally, an analysis of ChEMBL data revealed that only a small fraction of bioactive 1,5-DS-T compounds were synthesized via the Ugi-azide multicomponent reactions, a versatile and modular synthetic approach central to our research group. This highlights a valuable opportunity for future exploration and synthesis of novel 1,5-disubstituted-tetrazole-based scaffolds using Ugi-azide chemistry, with the aim of expanding chemical diversity and identifying new therapeutic agents to address unmet clinical challenges, including chemoresistant cancers.

## Supplementary Material



## Notes

Additional information about PCA analysis, sequence similarity analysis, docking results and In-house published structures (PDF)

## Abbreviations

1,5-DS-T: 1,5-disubstituted tetrazole
PCA: principal component analysis
CSR: cyclic system retrieval
PAP: phenylaminopyrimidine
CXCR3: chemokine receptor CXCR3
AMBR: antimicrobial bacterial resistance
QSAR: quantitative structure–activity relationship
MW: molecular weight
RTB: rotatable bonds
HBA: hydrogen bond acceptors
HBD: hydrogen bond donors
TPSA: topological polar surface area
HA: heavy atoms
LogP: octanol/water partition coefficient
NPR: normalized principal moment of inertia
WCSS: within-cluster sum of squares
MMPA: matched molecular pair analysis
CSFPs: cumulative scaffold frequency plots
FDA: Food and Drug Administration
LE: ligand efficiency
UA-MCR: Ugi–azide multicomponent reaction
EMT: epithelial–mesenchymal transition
RMSD: root-mean-square deviation
