# Supplementary material for: 1,5-Disubstituted Tetrazoles as Promising Anticancer Resistance Agents: A Chemoinformatic Characterization
Source: ACS Omega. 2026 May 20;11(21):31066–80. doi: 10.1021/acsomega.6c00605 (PMC13234678; doi:10.1021/acsomega.6c00605)
Supplement: Supplementary file 1 [file ao6c00605_si_001.pdf]

SUPPORTING INFORMATION

1,5-Disubstituted tetrazoles as promising anticancer resistance agents: A chemoinformatic Characterization

Camila Garibay-Manríquez[†], *Erik Díaz-Cervantes*[‡], *Luis Chacón-García*[†], *Karina Martínez-Mayorga*[€], *Carlos J. Cortés-García*[†]

[†]Laboratorio de Diseño Molecular, Instituto de Investigaciones Químico-Biológicas, Universidad Michoacana de San Nicolás de Hidalgo, Ciudad Universitaria 58030, Morelia, Michoacán, México.

[‡]Departamento de Alimentos, Centro Interdisciplinario del Noreste, Universidad de Guanajuato, 37975 Tierra Blanca, Guanajuato, México.

[€]Instituto de Química, Unidad Mérida, Universidad Nacional Autónoma de México, Carretera Mérida-Tetiz Km. 4.5, Ucú, Yucatán 97357, México.

* Corresponding authors.

kmtzm@unam.mx, jesus.cortes@umich.mx

TABLE OF CONTENTS

FIGURES

Figure S1. Percentage of explained variance of the bioactive chemical space of 1,5-DS-T……………………………………………………………..……………………………………………S3

Figure S2. Sequence similarity analysis of CCR3 using the NCBI BLAST tool to identify homologous proteins.……………………………………………………………………………………………..S4

Figure S3. Structural models and pocket analysis of CCR3 and CXCR3. (a) CCR3–CCL24 complex and (b) its fragmented binding site composed of multiple subpockets. (c) CXCR3–CXCL10 complex and (d) its single, continuous binding pocket identified by fpocket………………………………………………………………S6

Figure S4. Front and bottom views of IH-114 within the CXCR3 binding site………………...…………….. S7

Figure S5. Published In-house compounds Part 1……………………………………………………..……S8

Figure S6. Published In-house compounds Part 2……………………………………………………………S9

TABLES

Table S 1. Docking results of the ten compounds with the lowest total energy and lowest ligand efficiency……………………………………………………………………………………………………S5

Table S 2. Results of pockets identification and pockets description in FPocketWeb………………………...S5

Table S 3. Results of affinity prediction from Boltz-2……………………………………………………S10

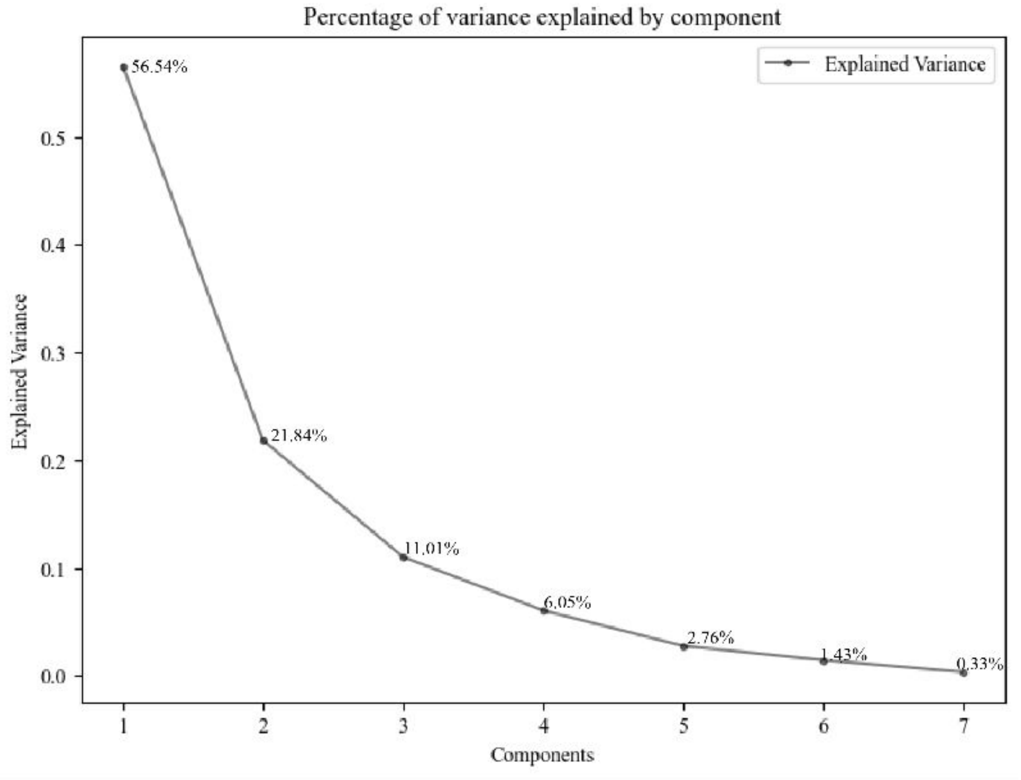

Figure S 1. Percentage of explained variance of the bioactive chemical space of 1,5-DS-T.

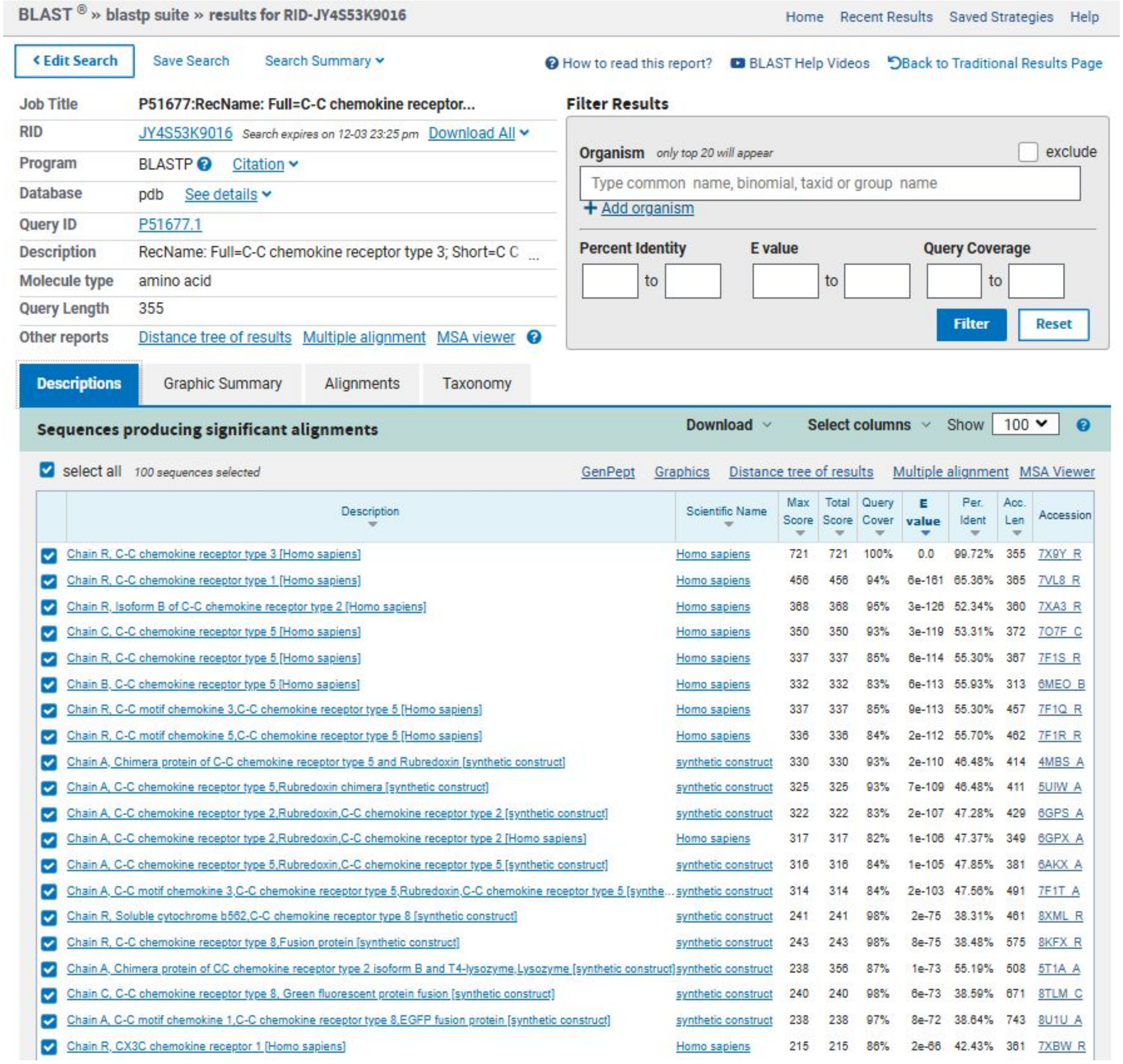

BLAST ® » blastp suite » results for RID-JY4S53K9016

Home Recent Results Saved Strategies Help

‹ Edit Search Save Search Search Summary ⌄ How to read this report? BLAST Help Videos Back to Traditional Results Page

Job Title	P51677:RecName: Full=C-C chemokine receptor...
RID	JY4S53K9016 Search expires on 12-03 23:25 pm Download All
Program	BLASTP Citation
Database	pdb See details
Query ID	P51677.1
Description	RecName: Full=C-C chemokine receptor type 3; Short=C C ...
Molecule type	amino acid
Query Length	355
Other reports	Distance tree of results Multiple alignment MSA viewer

Filter Results

Organism only top 20 will appear exclude

Type common name, binomial, taxid or group name

+ Add organism

Percent Identity to E value to Query Coverage to

Filter Reset

Descriptions Graphic Summary Alignments Taxonomy

Sequences producing significant alignments Download Select columns Show 100

select all 100 sequences selected GenPept Graphics Distance tree of results Multiple alignment MSA Viewer

Description	Scientific Name	Max Score	Total Score	Query Cover	E value	Per. Ident	Acc. Len	Accession
Chain R, C-C chemokine receptor type 3 [Homo sapiens]	Homo sapiens	721	721	100%	0.0	99.72%	355	7X9Y_R
Chain R, C-C chemokine receptor type 1 [Homo sapiens]	Homo sapiens	456	456	94%	6e-161	65.36%	365	7VL8_R
Chain R, Isoform B of C-C chemokine receptor type 2 [Homo sapiens]	Homo sapiens	368	368	95%	3e-126	52.34%	360	7XA3_R
Chain C, C-C chemokine receptor type 5 [Homo sapiens]	Homo sapiens	350	350	93%	3e-119	53.31%	372	7O7F_C
Chain R, C-C chemokine receptor type 5 [Homo sapiens]	Homo sapiens	337	337	85%	6e-114	55.30%	367	7F1S_R
Chain B, C-C chemokine receptor type 5 [Homo sapiens]	Homo sapiens	332	332	83%	6e-113	55.93%	313	6MEO_B
Chain R, C-C motif chemokine 3,C-C chemokine receptor type 5 [Homo sapiens]	Homo sapiens	337	337	85%	9e-113	55.30%	457	7F1Q_R
Chain R, C-C motif chemokine 5,C-C chemokine receptor type 5 [Homo sapiens]	Homo sapiens	336	336	84%	2e-112	55.70%	462	7F1R_R
Chain A, Chimera protein of C-C chemokine receptor type 5 and Rubredoxin [synthetic construct]	synthetic construct	330	330	93%	2e-110	46.48%	414	4MBS_A
Chain A, C-C chemokine receptor type 5,Rubredoxin chimera [synthetic construct]	synthetic construct	325	325	93%	7e-109	46.48%	411	5UIW_A
Chain A, C-C chemokine receptor type 2,Rubredoxin,C-C chemokine receptor type 2 [synthetic construct]	synthetic construct	322	322	83%	2e-107	47.28%	429	6GPS_A
Chain A, C-C chemokine receptor type 2,Rubredoxin,C-C chemokine receptor type 2 [Homo sapiens]	Homo sapiens	317	317	82%	1e-106	47.37%	349	6GPX_A
Chain A, C-C chemokine receptor type 5,Rubredoxin,C-C chemokine receptor type 5 [synthetic construct]	synthetic construct	316	316	84%	1e-105	47.85%	381	6AKX_A
Chain A, C-C motif chemokine 3,C-C chemokine receptor type 5,Rubredoxin,C-C chemokine receptor type 5 [synthe...	synthetic construct	314	314	84%	2e-103	47.56%	491	7F1T_A
Chain R, Soluble cytochrome b562,C-C chemokine receptor type 8 [synthetic construct]	synthetic construct	241	241	98%	2e-75	38.31%	461	8XML_R
Chain R, C-C chemokine receptor type 8,Fusion protein [synthetic construct]	synthetic construct	243	243	98%	8e-75	38.48%	575	8KFX_R
Chain A, Chimera protein of CC chemokine receptor type 2 isoform B and T4-lysozyme,Lysozyme [synthetic construct]	synthetic construct	238	356	87%	1e-73	55.19%	508	5T1A_A
Chain C, C-C chemokine receptor type 8, Green fluorescent protein fusion [synthetic construct]	synthetic construct	240	240	98%	6e-73	38.59%	671	8TLM_C
Chain A, C-C motif chemokine 1,C-C chemokine receptor type 8,EGFP fusion protein [synthetic construct]	synthetic construct	238	238	97%	8e-72	38.64%	743	8U1U_A
Chain R, CX3C chemokine receptor 1 [Homo sapiens]	Homo sapiens	215	215	86%	2e-66	42.43%	361	7XBW_R

Figure S 2. Sequence similarity analysis of CCR3 using the NCBI BLAST tool to identify homologous proteins.

Table S 1. Docking results of the ten compounds with the lowest total energy and lowest ligand efficiency.

Compound	Electro	LE	VdW	Hbond	E-Total
IH-114	-0.22	-4.46	108.00	-7.99	-201.01
IH-115	-0.15	-4.65	102.77	-6.77	-199.92
IH-86	1.33	-4.64	135.05	-9.43	-199.39
IH-94	1.03	-4.39	149.96	-2.65	-197.44
IH-90	0.43	-4.37	207.67	-12.67	-196.67
IH-33	-0.09	-4.63	93.11	-5.60	-189.78
IH-80	0.00	-4.87	122.90	-10.34	-189.78
IH-88	-0.21	-4.21	112.03	-12.84	-189.66
IH-32	-0.23	-4.86	186.30	-7.20	-189.42
IH-134	0.00	-4.61	84.26	-3.47	-188.93

Table S 2. Results of pockets identification and pockets description in FPocketWeb.

Chemokine receptor	# Of predicted pockets	Pocket	Score	Volume	Polar SA	Apolar SA	Hydrophobicity score	Polarity score
CCR3	32	4	0.65	451.74	0	12.84	45.8	6
		5	0.59	513.44	0	14.61	62.63	4
		15	0.34	318.75	0	8.06	51.77	4
		22	0.30	167.08	0	8.68	51.42	6
		Average	NA	1451.01	0	44.19	NA	NA
CXCR3	14	1	1.73	1765.47	0	65.36	25.85	21

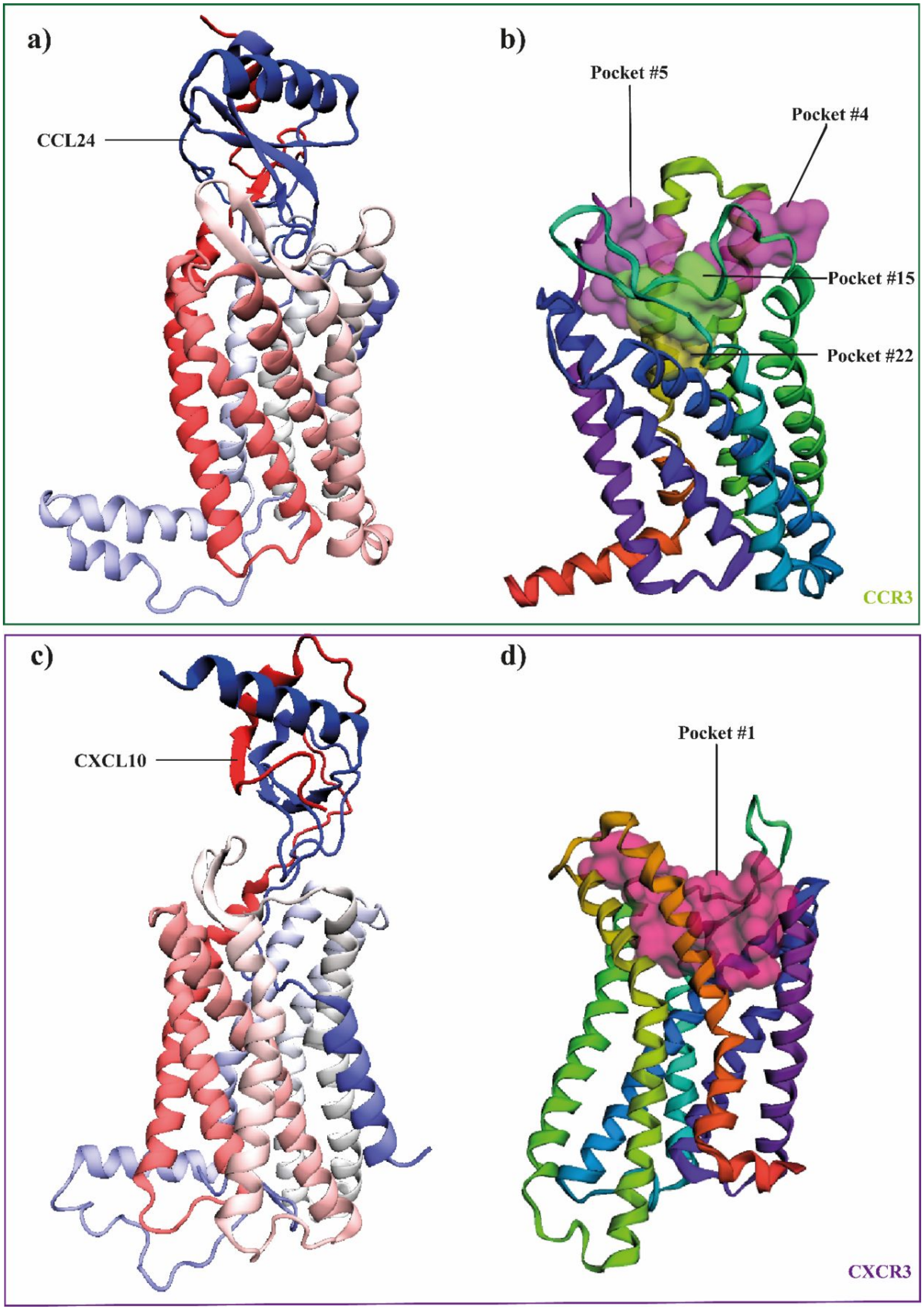

Figure S3. Structural models and pocket analysis of CCR3 and CXCR3. (a) CCR3–CCL24 complex and (b) its fragmented binding site composed of multiple subpockets. (c) CXCR3–CXCL10 complex and (d) its single, continuous binding pocket identified by fpocket.

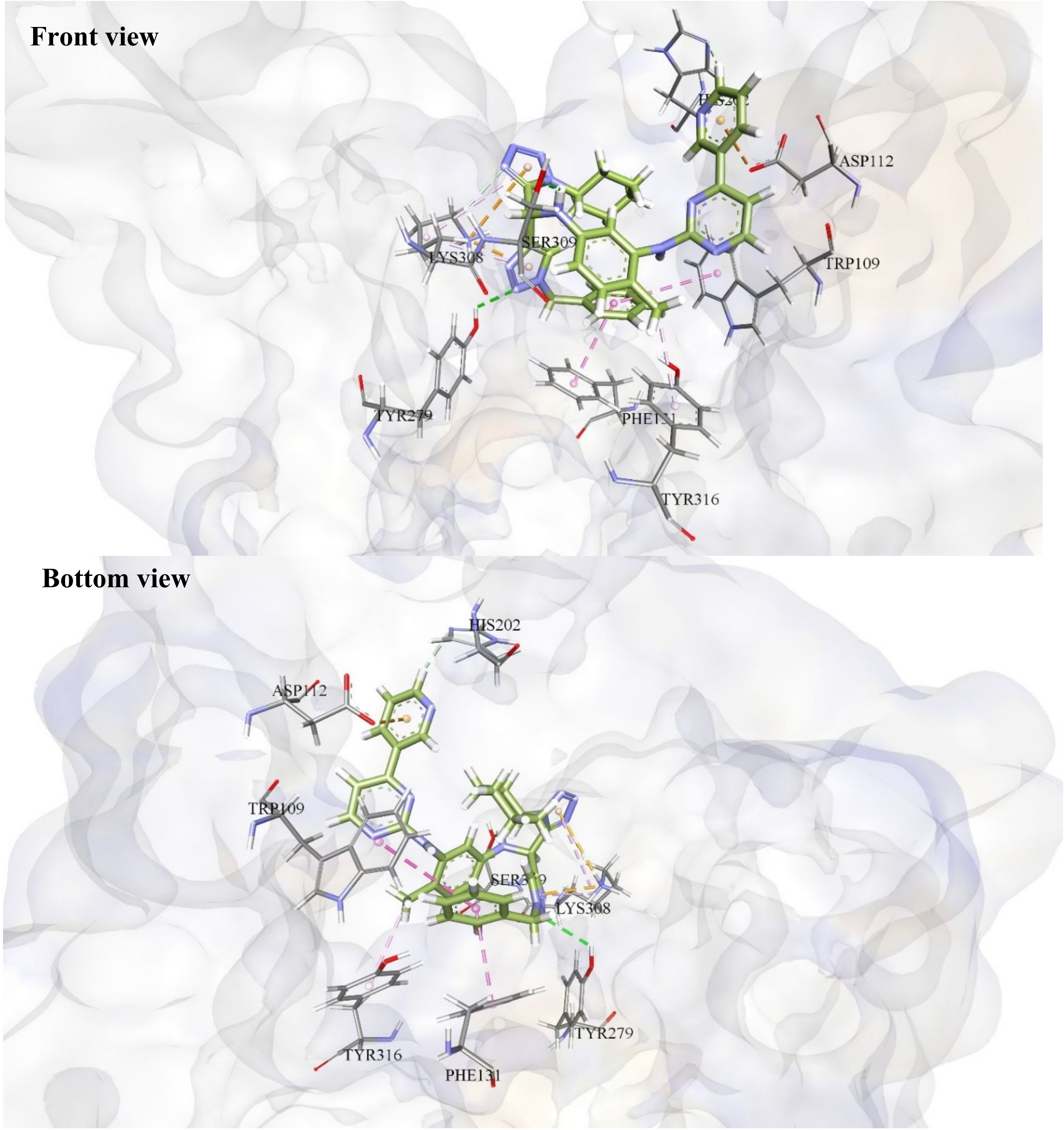

Figure S4. Front and bottom views of IH-114 within the CXCR3 binding site.

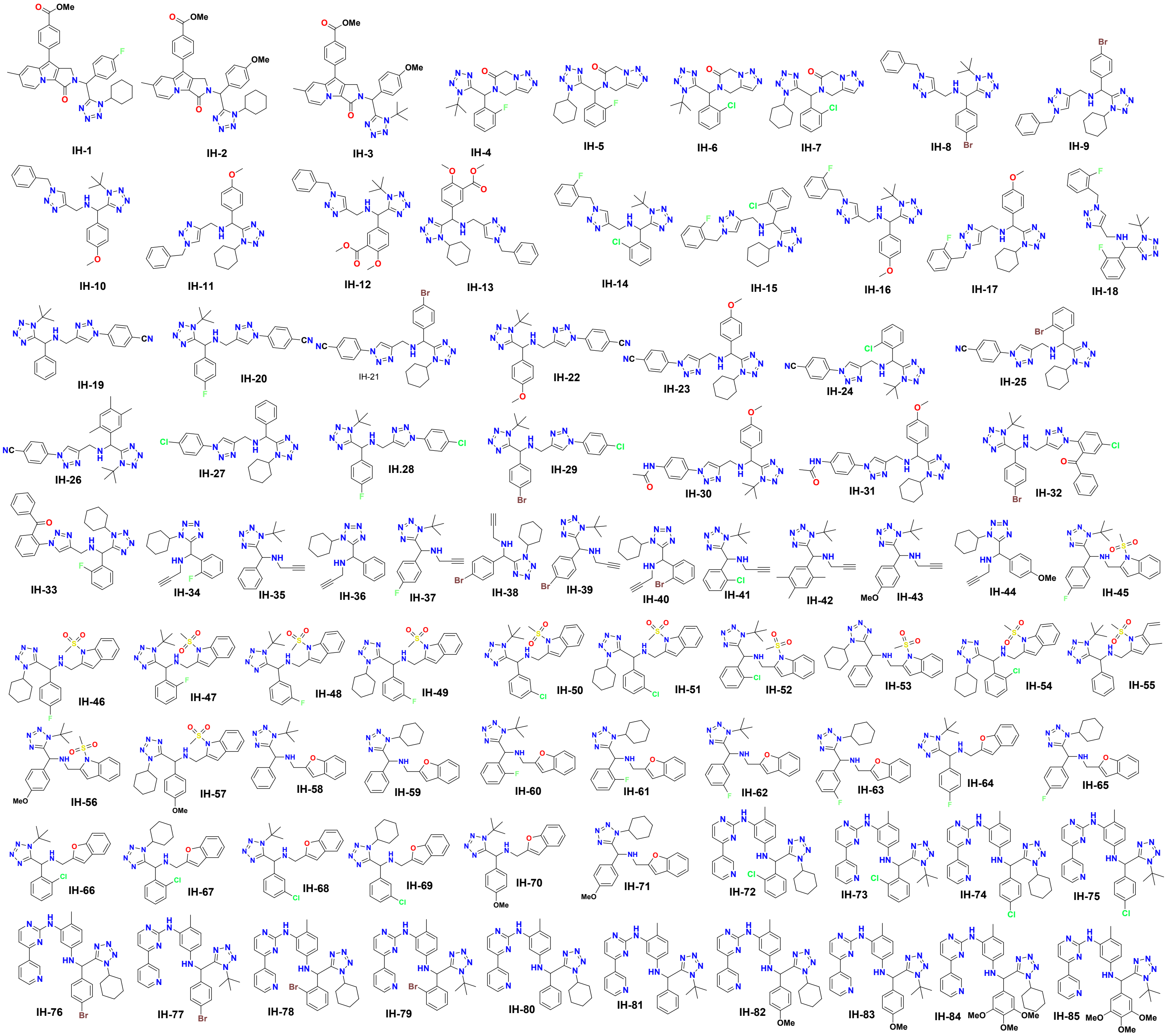

Figure S5. Published in-house compounds Part 1.

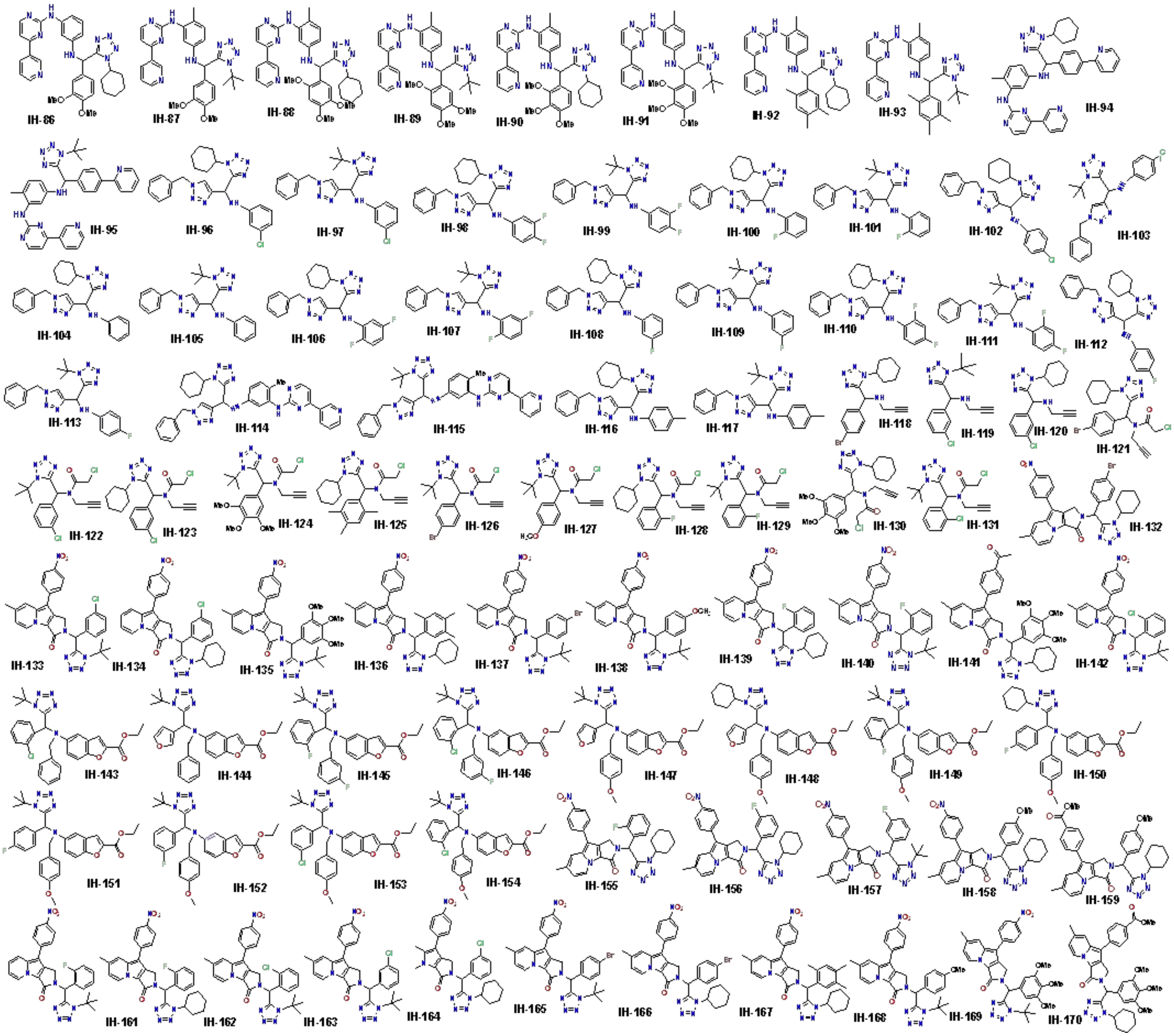

Figure S6. Published in-house compounds Part 2.

Table S 3. Results of affinity prediction from Boltz-2.

Compound	Affinity probability [a]	Boltz affinity score [b]	Measured pIC50 (kcal/mol) [c]	Measured IC50 (µM) [d]	Confidence Score [f]
AMG-487	0.61	-1.12	9.71	0.08	0.80
IH-114	0.58	-0.43	7.59	0.37	0.83
IH-39	0.48	1.71	5.85	51.00	0.81
IH-25	0.37	0.84	7.03	6.90	0.79

[a] Probability that the ligand is a binder: 0-1

[b] -3 (strong binder); 0 (moderate binder); 2 (weak binder/decoy)

[c] (6-y) * 1.364 where y es the model's prediction of affinity

[d] If the affinity score is equal to -3 then IC50 = 10-9 M; equal to 0 then IC50 = 10-6 M; equal to 2 then IC50 = 10-4 M

[f] Corresponds to 0.8 * complex_plddt + 0.2 * iptm (ptm for single chains)
